# Genome-wide association study in Alzheimer’s disease: a bibliometric and visualization analysis

**DOI:** 10.3389/fnagi.2023.1290657

**Published:** 2023-11-29

**Authors:** Junyao Zhang, Yinuo Wang, Yingying Zhang, Junyan Yao

**Affiliations:** ^1^Department of Anesthesiology, Shanghai General Hospital, Shanghai Jiao Tong University School of Medicine, Shanghai, China; ^2^Department of Anesthesiology and Critical Care Medicine, Shanghai East Hospital, Tongji University School of Medicine, Shanghai, China

**Keywords:** Alzheimer’s disease, genome-wide association study, bibliometric, CiteSpace, VOSviewer

## Abstract

**Background:**

Thousands of research studies concerning genome-wide association studies (GWAS) in Alzheimer’s disease (AD) have been published in the last decades. However, a comprehensive understanding of the current research status and future development trends of GWAS in AD have not been clearly shown. In this study, we tried to gain a systematic overview of GWAS in AD by bibliometric and visualization analysis.

**Methods:**

The literature search terms are: (“genome-wide analysis” or “genome-wide association study” or “whole-genome analysis”) AND (“Alzheimer’s Disease” or “Alzheimer Disease”). Relevant publications were extracted from the Web of Science Core Collection (WoSCC) database. Collected data were further analyzed using VOSviewer, CiteSpace and R package Bibliometrix. The countries, institutions, authors and scholar collaborations were investigated. The co-citation analysis of publications was visualized. In addition, research hotspots and fronts were examined.

**Results:**

A total of 1,350 publications with 59,818 citations were identified. The number of publications and citations presented a significant rising trend since 2013. The United States was the leading country with an overwhelming number of publications (775) and citations (42,237). The University of Washington and Harvard University were the most prolific institutions with 101 publications each. Bennett DA was the most influential researcher with the highest local H-index. *Neurobiology of Aging* was the journal with the highest number of publications. Aβ, tau, immunity, microglia and DNA methylation were research hotspots. Disease and causal variants were research fronts.

**Conclusion:**

The most frequently studied AD pathogenesis and research hotspots are (1) Aβ and tau, (2) immunity and microglia, with *TREM2* as a potential immunotherapy target, and (3) DNA methylation. The research fronts are (1) looking for genetic similarities between AD and other neurological diseases and syndromes, and (2) searching for causal variants of AD. These hotspots suggest noteworthy directions for future studies on AD pathogenesis and genetics, in which basic research regarding immunity is promising for clinical conversion. The current under-researched directions are (1) GWAS in AD biomarkers based on large sample sizes, (2) studies of causal variants of AD, and (3) GWAS in AD based on non-European populations, which need to be strengthened in the future.

## Introduction

Alzheimer’s disease (AD) is the most common cause of dementia, with tremendous economic and social burden. About 6.7 million Americans living with AD in 2023 are 65 years and older, and as the aging population increases, the prevalence of dementia is predicted to increase to 131.5 million in 2050 (Anonymous, 2023, p. 1598–1695). With memory and cognitive decline, AD can have a significant negative impact on the patient and family (Hsiao et al., 2018). However, there is still no satisfactory treatment for diagnosed AD (Golde, 2022). Therefore, identifying risk factors and susceptible populations for AD is crucial for targeted interventions.

AD is a genetically complex disorder with an apparent hereditary predisposition, and the heritability factors account for 58–79% of the risk of late-onset AD, and up to 92–100% of the risk of early-onset AD (Sims et al., 2020; Ayodele et al., 2021). Therefore, identifying genetic characteristics not only is essential for fundamentally understanding AD etiology, but also provides with the possibility of early intervention for susceptible populations. Linkage studies have long established *APP*, *PSEN1* and *PSEN2* to cause early-onset AD, while early-onset form only accounts for less than 5% of all AD cases (Ikram and Decarli, 2012). The discovery of *APOEε4* in 1993 denoted the first gene to increase susceptibility for the more common late-onset AD, and *APOEε4* remained the only robustly replicated gene for late-onset AD for almost two decades (Bertram et al., 2010; Serrano-Pozo et al., 2021). However, finding more AD risk variants was difficult due to the limitations of research technology at that time.

In recent years, technological advances have given rise to genome-wide association studies (GWAS) in AD (Andrews et al., 2020). By using high-throughput genotyping and next-generation sequencing, GWAS has made it possible to uncover more AD risk variants (Xiao et al., 2022). GWAS has increased statistical power by adding clinically or pathologically diagnosed cases and controls, and has facilitated the discovery of DNA sequence variations across the human genome (Andrews et al., 2020). By GWAS, single nucleotide polymorphisms (SNPs) that are highly associated with the disease can be identified (Gibson, 2010). Progress in GWAS and global cooperation of genome projects started a new era of exploring AD genetic characteristics and helped us to predict AD occurrence better. Since the first GWAS in AD emerged in 2007, GWAS has facilitated the exploration of the genetic architecture underlying amyloid-β (Aβ) and tau, and implicated a host of genetic variants associated with Aβ and tau biological processing (Khani et al., 2022). Meanwhile, a large number of genes involved in physiopathologic processes including cholesterol metabolism (*APOE*, *CLU*, *ABCA7* and *SORL1*), immune response (*CR1*, *CD33*, *MS4A*, *CLU*, *ABCA7* and *EPHA1*), endocytosis (*BIN1*, *PICALM*, *CD2AP*, *EPHA1* and *SORL1*) have been proven to be associated with the risk of AD (Ikram and Decarli, 2012; Karch and Goate, 2015). However, among these gene-indicated mechanisms, which are most critical in the AD process remains unknown. In addition, research interests were constantly changing with the development of the discipline. Branches of GWAS such as single-cell genomics, transcriptomics, metabolomics, epigenomics, and the exploration of gene-disease causation have been applied to the genetic exploration of AD. However, the current cutting-edge research directions have not yet been revealed. Therefore, a comprehensive literature analysis of GWAS for the AD field is necessary.

Bibliometrics is a reliable means for literature analysis. It allows quantitative description for publications of a specific field using mathematical and statistical methods, and enables visualization of bibliometric statistics by CiteSpace, VOSviewer and R software. It helps researchers identify research trends, hotspots and critical cooperation networks that collectively guide academic decisions (Ma and Ho, 2016; Chen, 2017). In this study, we drew a whole picture of GWAS in AD from the first publication to 2022 in a bibliometric way, to provide hints for future explorations in this field.

## Methods

### Data source and search strategies

**Figure 1:**
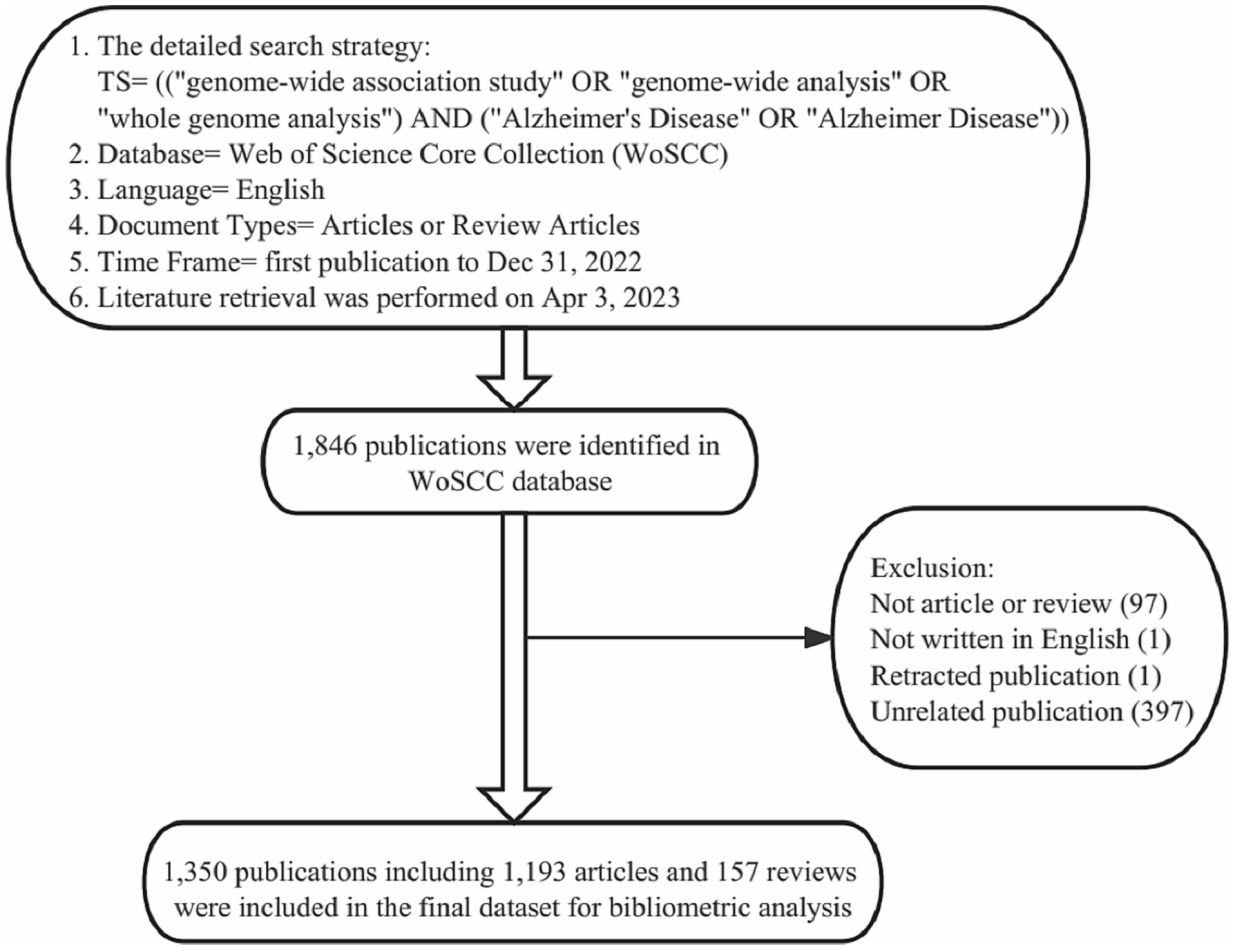
Details of the literature searching and data filtrating. 1,846 publications on the topic of GWAS in AD were identified in the WoSCC database, and a total of 496 publications were manually excluded. Finally, 1,350 publications were included in the final dataset for bibliometric and visualization analysis.

A comprehensive search was performed within the Web of Science Core Collection (WoSCC) database (Clarivate Analytics, Philadelphia, PA, United States) using the following search strategy: TS = (“genome-wide analysis” or “genome-wide association study” or “whole-genome analysis”) AND (“Alzheimer’s Disease” or “Alzheimer Disease”), document type = (article or review), and language = English. The time frame was limited from January 1, 2007 to December 31, 2022. All data searches and retrievals were completed in a single day on April 3, 2023, to minimize the bias led by database updates (Figure 1).

### Data extraction and screening

All bibliographical information of publications, including publication year, title, author’s names, affiliations, nationalities, abstracts, keywords, journal names and citation numbers, were recorded and then converted to text format. Subsequently, the data was imported into Microsoft Excel 2019 (Microsoft Corporation). Non-English publications and duplicate publications were removed. Then two researchers (Junyao Zhang and Yinuo Wang) independently examined and manually excluded the publications unrelated to the topic, 1,221 publications were agreed to meet the inclusion criteria. Then, the third researcher (Yingying Zhang) examined the parts where the other two researchers disagreed (234 publications) and made a final assessment to reach a consensus among researchers. In the end, 1,350 publications (including 1,193 articles and 157 reviews) were included in the final dataset (Figure 1).

### Bibliometric analysis

The final dataset was imported into bibliometric software. VOSviewer (version 1.6.18) was used for co-authorship analysis of countries, institutions and researchers, and also used for keyword co-occurrence analysis. CiteSpace (version 5.8.R3) was used for publication co-citation analysis, knowledge base analysis, knowledge flow analysis and burst keyword analysis. R package Bibliometrix (version 4.2.1) was used for the authors’ local H-index analysis. In addition, the Microsoft Excel 2019 (Microsoft Corporation) software was used to present global trends of annual publications and citations. SCImago Graphica (version beta 1.0.21) was used for geographic visualization in country co-authorship analysis.

## Results

### Analysis of global publication output and citation trend

**Figure 2:**
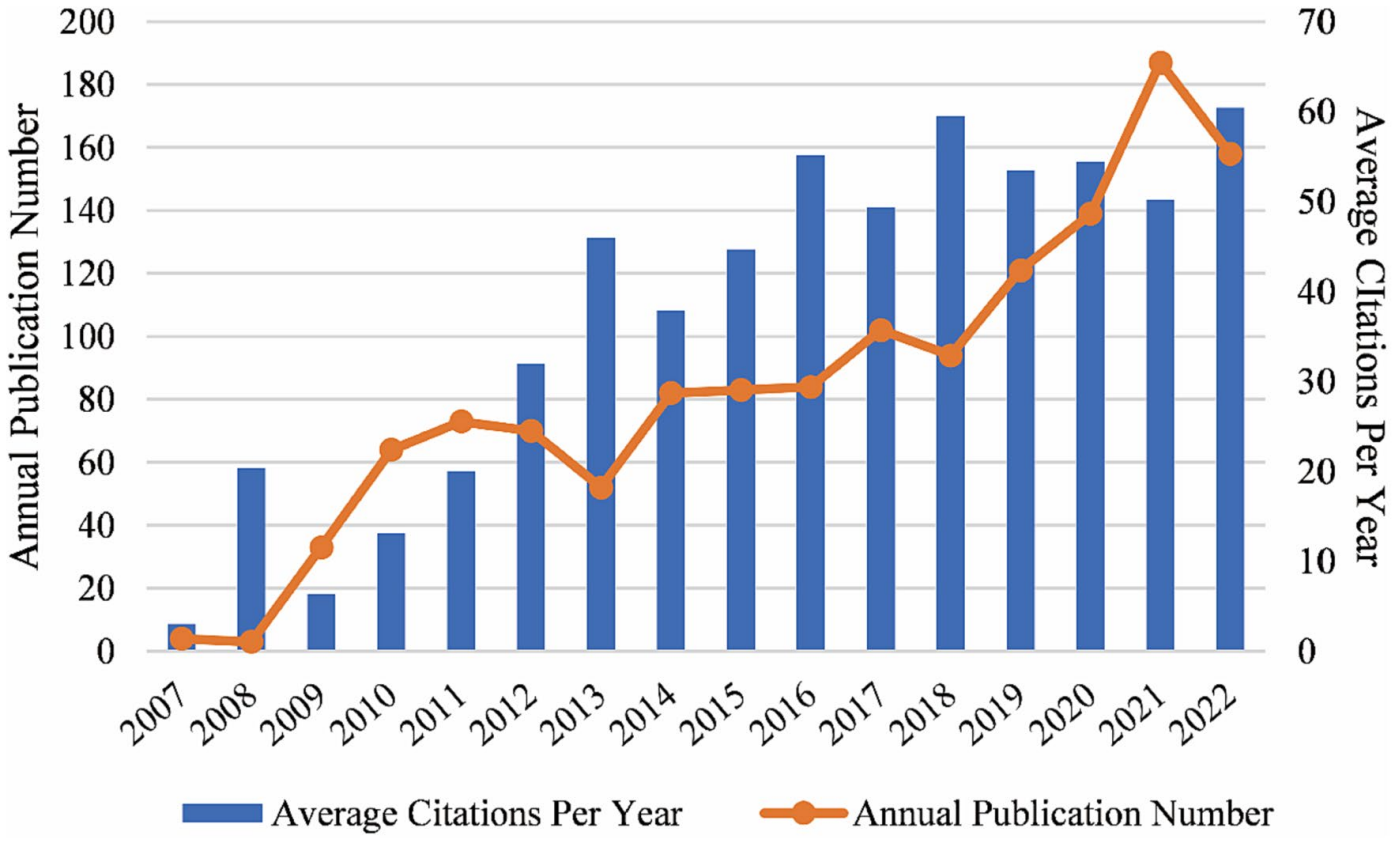
Global trends of annual publications and citations on the topic of GWAS in AD from the first publication to 2022, generated by Excel. From 2007 to the end of 2022, the number of publications and citations in this field was generally on the rise.

All the 1,350 publications (including 1,193 articles and 157 reviews) were cited 59,818 times from the first publication in 2007 to the end of 2022. The trend of annual publication and citation numbers was generally on the rise. The annual publication number increased rapidly after 2013 and peaked in 2021 with 187 publications. The average number of citations per year increased and maintained at a high level after 2013 (Figure 2).

### Distribution of countries and regions

**Figure 3:**
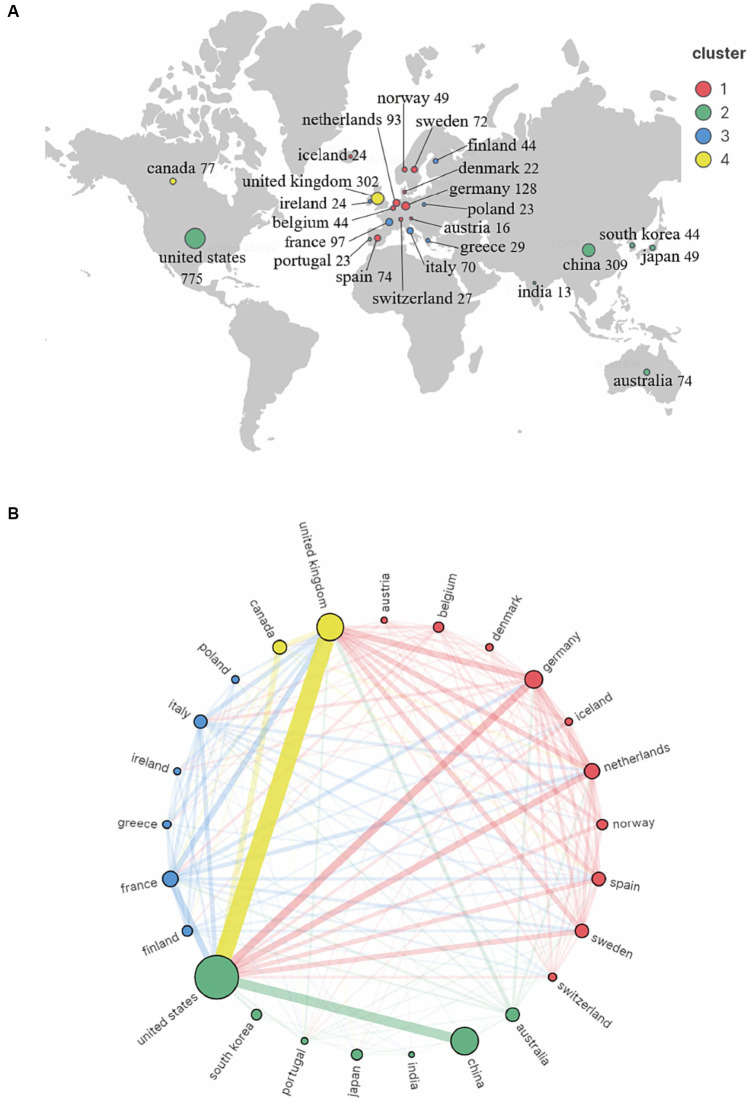
Co-authorship country and region analysis on GWAS in AD from first publication to 2022. **(A)** Geographic map of country and region distribution generated by VOSviewer and Scimago Graphica. The countries/regions that cooperate extensively with each other were assigned to the same cluster. **(B)** Cordal graph of country and region cooperation network generated by VOSviewer and Scimago Graphica. 28 countries or regions met the criteria that publishing more than 8 publications were grouped into 4 clusters by their cooperations. The node size indicates the cooperative activity, the larger the node, the more cooperation the country or region has with others.

Seventy countries and regions participated in the publication of GWAS in AD. When ranked by the number of publications, the United States was the most productive country with 775 publications cited 42,237 times, followed by China with 309 publications cited 7,259 times, and the United Kingdom with 302 publications cited 25,311 times. The map indicating the contributions of countries or regions in this field is shown in Figure 3A. The top 10 countries with the most significant number of publications are listed in Table 1.

**Table 1:** List of top 10 countries/regions.

Country/Region	Publications	Citations	Total Link Strength^*^
The United States	775	42,237	981
China	309	7,259	207
The United Kingdom	302	25,311	810
Germany	128	15,730	509
France	97	14,893	473
Netherlands	93	12,780	485
Canada	77	12,992	272
Spain	74	11,550	347
Australia	74	4,839	261
Sweden	72	8,481	356

^*^Total link strength indicates the cooperative activity of the country or region.

A country co-authorship map is shown in Figure 3B. The United States was the most active country and extensively cooperated with the United Kingdom, China and other countries. European countries also worked closely with each other and contributed a lot in the field of GWAS in AD.

### Analysis of the most productive institutions

Overall, a total of 1,819 institutions performed GWAS in AD. As presented in Table 2, both the University of Washington and Harvard University topped the list with 101 publications each, closely followed by Columbia University with 92 publications. Harvard University was the research institution with the highest total citations (6,851 citations), while the University of Washington and Boston University followed with a total citation of 5,817 and 4,715, respectively.

**Table 2:** List of top 10 institutions.

Institution	Publications	Citations	Total link strength^*^
Harvard University	101	6,851	582
The University of Washington	101	5,817	685
Columbia University	92	4,127	606
Boston University	80	4,715	588
The University of Pennsylvania	79	4,303	590
Rush University	71	4,595	535
The University of Miami	67	4,364	509
Indiana University School of Medicine	67	2,752	348
The Mayo Clinic	64	4,550	390
Qingdao University	62	869	84

^*^Total link strength indicates the cooperative activity of the institution.

**Figure 4:**
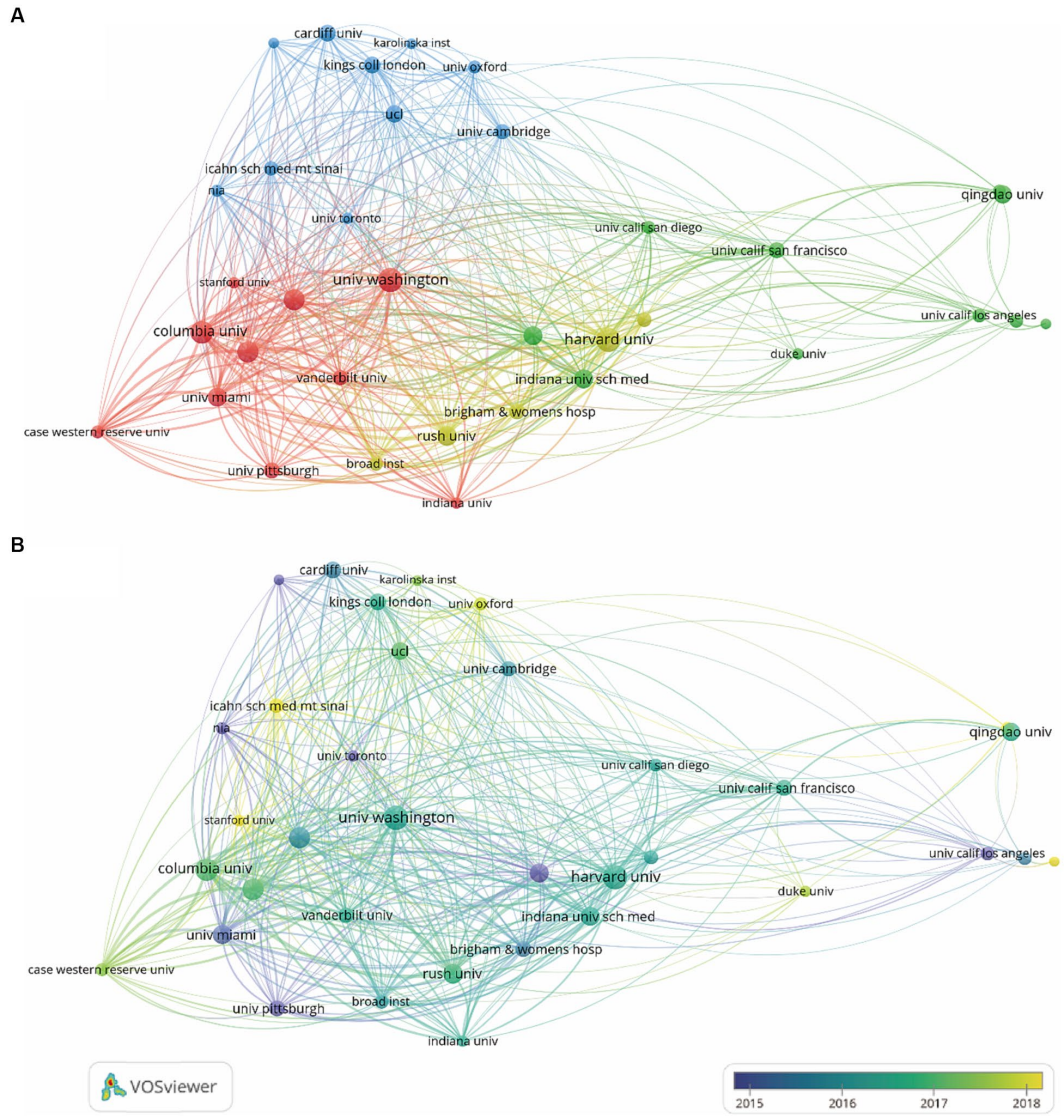
Co-authorship institution analysis on the topic of GWAS in AD from first publication to 2022. **(A)** Institution co-authorship network map generated by VOSviewer. 35 institutions that met the standard of having more than 25 publications were included and grouped into 4 clusters by their cooperations. The node size denotes the cooperative activity, the larger the node, the more cooperation the institutions had with others. **(B)** Institution co-authorship overlay map generated by VOSviewer. The node color indicates the average publication time of the institution, the lighter the color of the node, the more recent the average publication time of the institution.

An institution co-authorship map is shown in Figure 4A. Institutions in the United States, like Harvard University, the University of Washington, Columbia University, Rush University and the University of Miami were most active in collaborating with other institutions.

According to the overlay map generated by VOSviewer, the Mayo Clinic, the University of Pittsburgh, the University of Toronto, the University of Miami and the University of California Los Angeles started working on GWAS in AD the earliest. Columbia University, Harvard University, Rush University, the University of Washington, the University of Cambridge and King’s College London published the most between 2016 and 2017. Research by Stanford University, the University of Oxford and Duke University were the institutions published the most after 2018 (Figure 4B).

### Analysis of the most influential researchers

**Figure 5:**
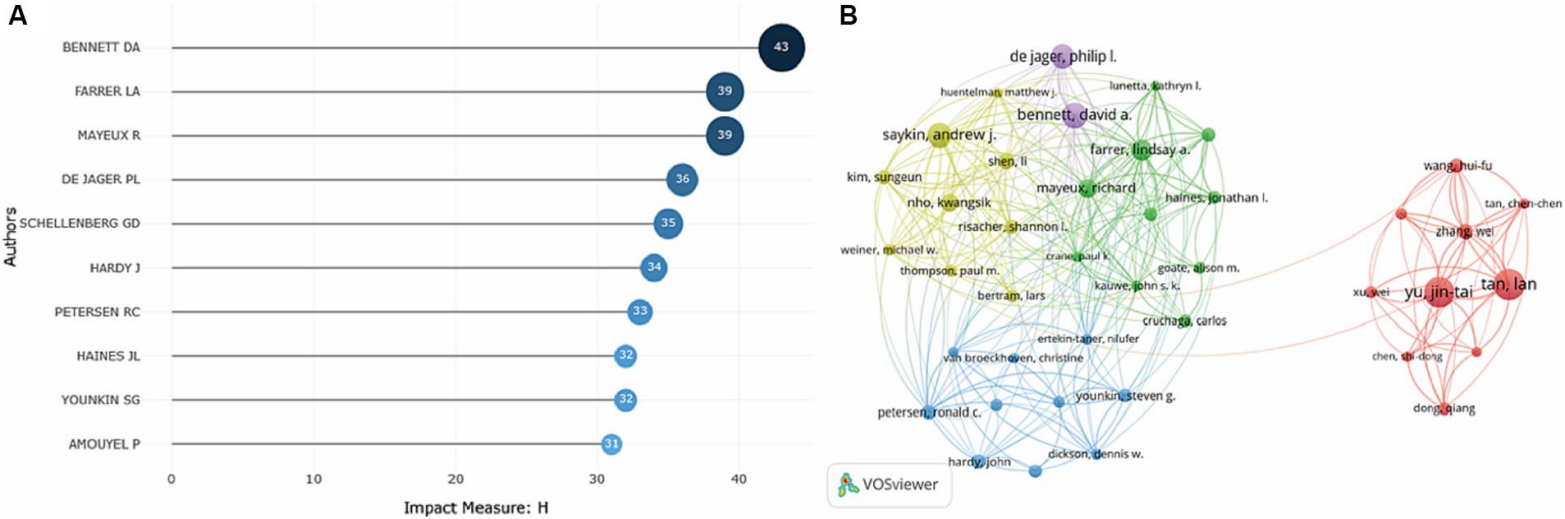
Analysis of the most influential researchers on the topic of GWAS in AD from first publication to 2022. **(A)** Ten researchers with the highest local H-index in this field were generated by the R package Bibliometrix. **(B)** Researcher co-authorship network map generated by VOSviewer. Researchers with at least 13 publications were included in the map, and a total of 50 researchers who met the criteria were grouped into 5 clusters. The node size denotes the cooperative activity, the larger the node, the more the researcher cooperates with others.

Local H-index refers to h publications cited at least h times for each paper in a certain field. It is an important index to comprehensively measure the quantity and quality of a researcher’s output. To investigate the most influential researchers, the researchers with the highest H-index were shown. As demonstrated in Figure 5A, Bennett DA, Farrer LA, Mayeux R and De Jager PL were the most influential researchers with high-quality publications in this field.

As indicated by the researcher co-authorship map, Bennett DA, De Jager PL and Saykin AJ were critical researchers and cooperated extensively with others. Researchers from China were in the red cluster, with strong collaborative relationships established with each other, while little collaboration between Chinese researchers and researchers in other countries was observed. And Yu JT and Tan L were influential researchers in this cluster (Figure 5B).

### Representative publications and knowledge bases

**Figure 6:**
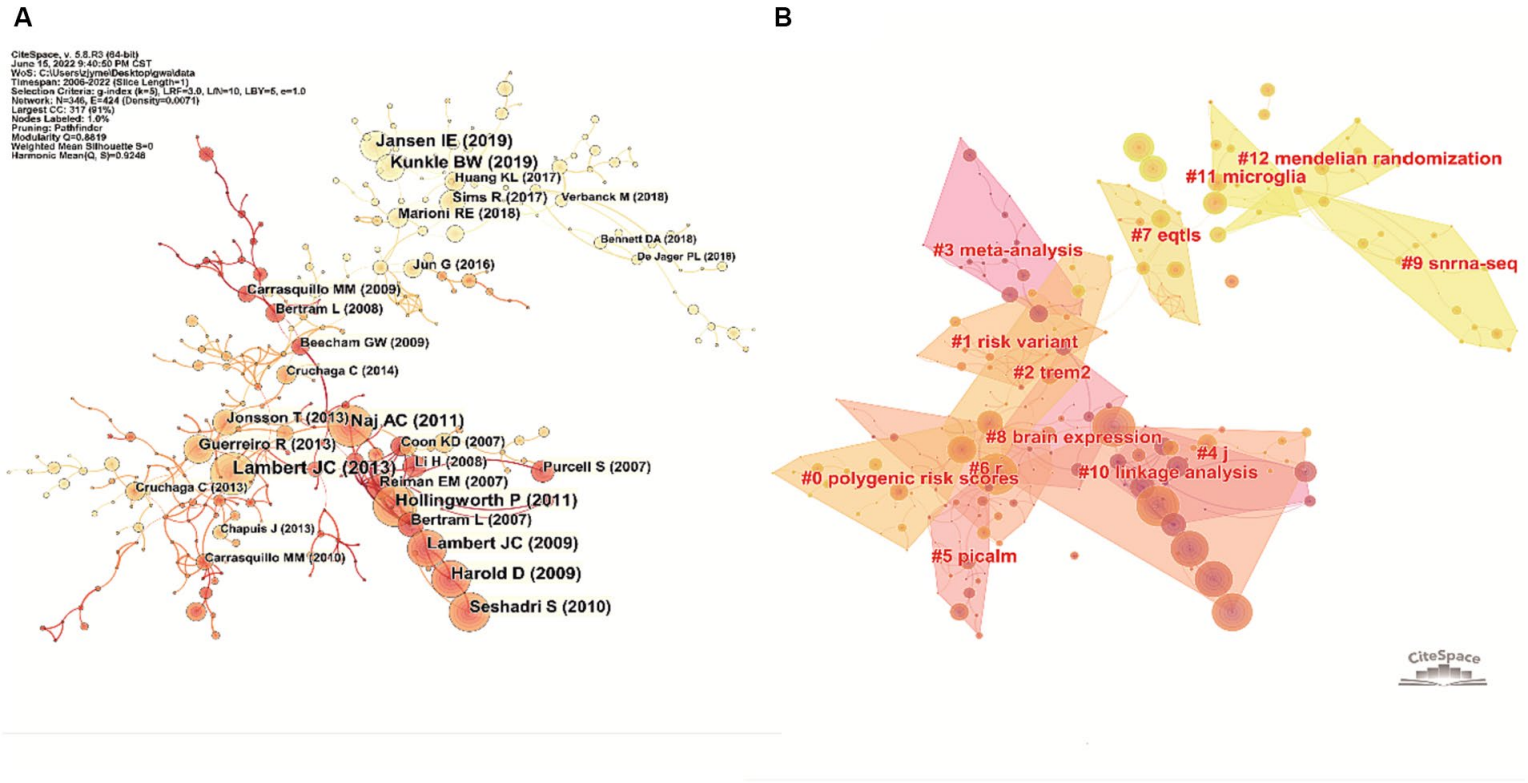
Analysis of the representative publications on the topic of GWAS in AD from first publication to 2022. **(A)** Co-citation network of the publications in this field generated by CiteSpace. The node size positively correlates with the corresponding publication’s total citation number. The color of the node indicates the publication time, the lighter the node color, the more recent the publication time. **(B)** Knowledge base map generated by clustering the co-cited publications. Publications in close co-citation relationships were divided into the same cluster, and tags representing the cluster’s knowledge base were extracted from publications’ titles.

According to the publication co-citation analysis in Figure 6A, Bertram et al. (2007), Harold et al. (2009), Lambert et al. (2009), Seshadri et al. (2010), Hollingworth et al. (2011), Naj et al. (2011), Guerreiro et al. (2013), and Lambert et al. (2013) were early publications in the field, cited numerous times, these publications could be regarded as roots of the area. Recent work including Jun (2016), Sims et al. (2017), Marioni (2018), Jansen et al. (2019), and Kunkle et al. (2019) were also cited a lot, although it has only been a short time since their publication. The top 10 most-cited publications are listed in Table 3.

**Table 3:** List of 10 most-cited publications.

Author/Year	Title
Lambert et al. (2013)	Meta-analysis of 74,046 individuals identifies 11 new susceptibility loci for Alzheimer’s disease
Harold et al. (2009)	Genome-wide association study identifies variants at *CLU* and *PICALM* associated with Alzheimer’s disease
Guerreiro et al. (2013) and Guerreiro et al. (2013)	*TREM2* variants in Alzheimer’s disease
Lambert et al. (2009)	Genome-wide association study identifies variants at *CLU* and *CR1* associated with Alzheimer’s disease
Hollingworth et al. (2011)	Common variants at *ABCA7*, *MS4A6A/MS4A4E*, *EPHA1*, *CD33* and *CD2AP* are associated with Alzheimer's disease
Naj et al. (2011)	Common variants at *MS4A4/MS4A6E*, *CD2AP*, *CD33* and *EPHA1* are associated with late-onset Alzheimer’s disease
Kunkle et al. (2019)	Genetic meta-analysis of diagnosed Alzheimer’s disease identifies new risk loci and implicates Aβ, tau, immunity and lipid processing
Jansen et al. (2019)	Genome-wide meta-analysis identifies new loci and functional pathways influencing Alzheimer’s disease risk
Seshadri et al. (2010) and Seshadri et al. (2010)	Genome-wide analysis of genetic loci associated with Alzheimer disease
Bertram et al. (2007)	Systematic meta-analysis of Alzheimer’s disease genetic association studies: the AlzGene database

In the knowledge base map, articles co-cited by another article had a close academic relationship and were therefore grouped into the same cluster. Tags that represent the knowledge base of the cluster were extracted from the titles of the publications. As demonstrated in Figure 6B, 11 crucial tags were extracted (after removing tags without academic meaning), including #polygenic risk scores, #risk variant, #*TREM2*, #meta-analysis, #*PICALM*, #eQTLs, #brain expression, #snRNA-seq, #linkage analysis, #microglia and #Mendelian randomization.

### Analysis of prolific journals and knowledge flow

As demonstrated in Table 4, *Neurobiology of Aging* had 105 publications and 2,910 total citations, followed by *J Alzheimer’s Disease* with 95 publications cited 1,561 times, *Plos One* with 49 publications cited 2,277 times, and *Alzheimer’s & Dementia* with 47 publications cited 1,805 times. Although *Nature Genetics* only had 22 publications, it received an overwhelming 11,327 citations.

**Table 4:** List of top 10 journals.

Journals	Publications	Citations
Neurobiology of Aging	105	2,910
J Alzheimer’s Disease	95	1,561
Plos One	49	2,277
Alzheimer’s and Dementia	47	1,805
Molecular Psychiatry	43	2,267
Translational Psychiatry	30	652
Scientific Reports	28	306
Molecular Neurobiology	27	628
Nature Genetics	22	11,327
Human Molecular Genetics	22	1,151

**Figure 7:**
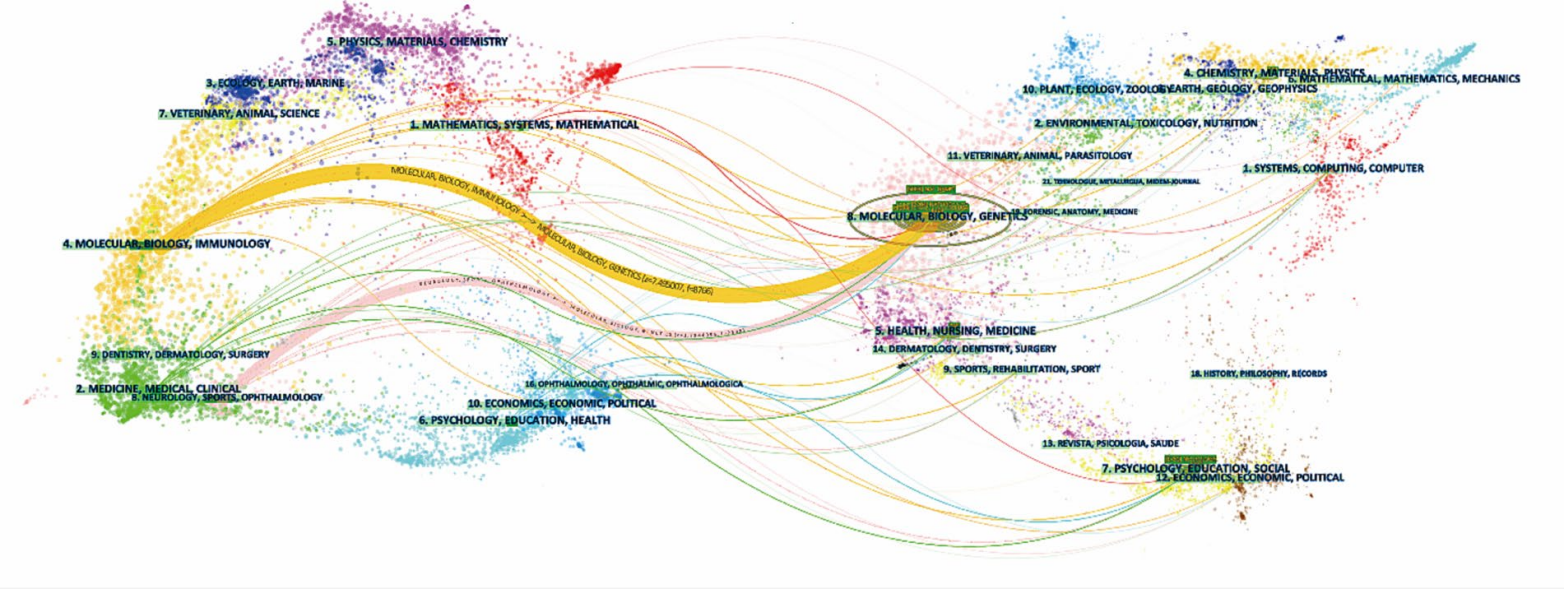
Knowledge flow overlay generated by CiteSpace. The cited journals are on the right half of the map, and the citing journals are on the left. The main citation paths were pointing from basic disciplines (molecular, biology and genetics) to basic research (molecular, biology, immunology) and clinical medicine (medicine, medical clinical).

To display the evolutionary relationship between citing and cited journals, the knowledge flow overlay map was generated (Chen, 2017). The dots on the left part of the map constitute the citing journals, and the dots on the right include the cited journal. Labels extracted from journal names are linked by lines pointing from the cited journals to the citing journals. As shown in Figure 7, the wide yellow line and the wide pink line were core citation paths, indicating publications in journals of basic science (molecular, biology and genetics) were mainly cited by publications in journals related to basic science (molecular, biology, immunology) and clinical medicine (medicine, medical clinical).

### Analysis of essential keywords and hotspots

**Figure 8:**
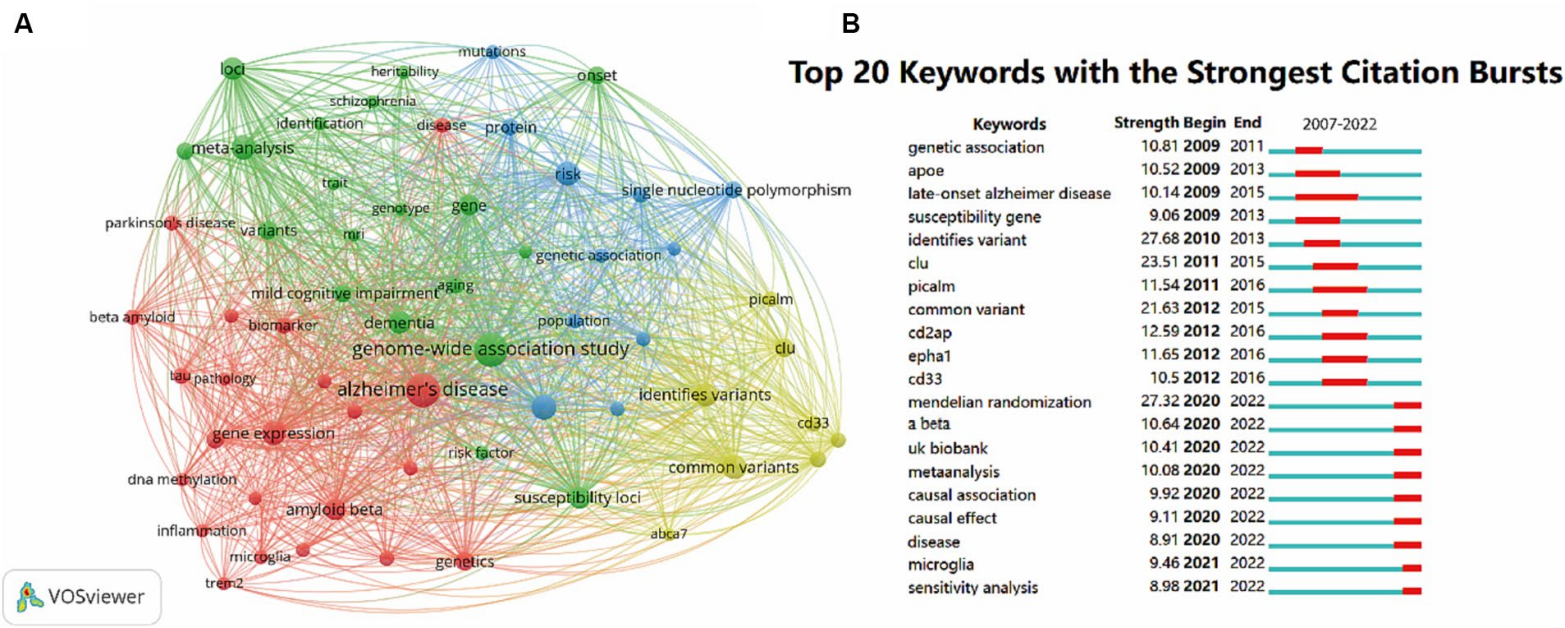
Co-occurrence analysis of global research on the topic of GWAS in AD from first publication to 2022. **(A)** Network visualization map keywords generated by VOSviewer. Keywords that occurred at least 30 times were included in the map, and a total of 60 keywords that met the criteria were divided into 4 clusters. The node size represents the keyword occurrence frequency. **(B)** The top 20 keywords with the strongest citation bursts by CiteSpace. The red segment indicates a sudden increase in the occurrence frequency of the keyword during this period.

A co-occurring keyword network based on 4,077 extracted keywords was generated to show the most frequently mentioned keywords. The keywords displayed on the map were divided into four clusters. GWAS terms were in the green cluster, and the pathogenesis of AD (#Aβ, #tau, #DNA methylation, #microglia, #gene expression, #inflammation) belonged to the red cluster. #genetic association, #protein, #SNP and #population were assigned to the blue cluster. While the most often mentioned genes, including #*PICALM*, #*CLU*, #*CD33* and #*ABCA7*, were shown in the yellow cluster (Figure 8A).

To visualize the dynamics of frontier topics in the field, the burst analysis of keywords from 2007 to 2022 was performed. The whole segments stand for the time span of each keyword, while the red segments represent sudden increases in the frequency of the keyword occurrence during this period. The burst strength is a statistical value calculated from the keyword occurrence frequency over time. The top 20 keywords with the strongest bursts are shown in Figure 8B. The keywords #genetic association, #*APOE*, #late-onset AD, #suseptibility gene, #identifies variant, #*CLU*, #*PICALM*, #common variant, #*CD2AP*, #*EPHA1*, #*CD33* had burst time before 2016. The keywords #Mendelian randomization, #Aβ, #UK Biobank, #meta-analysis, #causal association, #causal effect, #disease, #microglia and #sensitivity analysis has become popular since 2020 till now, suggesting the present research fever of the field.

## Discussion

### Research trend and current profile of GWAS in AD

We investigated 1,350 publications, including original articles and reviews on GWAS in the AD field between 2007 and 2022. Since several small GWAS opened the new era of AD research in 2007, the number of annual publications of GWAS in AD has been generally on the rise, indicating it is an academic concern. There were not many citations in the first few years, while the average citations per year increased and maintained at a high level after 2013 (Figure 2), which suggests that a growing number of high-quality, influential studies emerged. In 2013, a large-scale GWAS was conducted by the International Genomics of Alzheimer’s Project with 11 new AD-associated loci identified, it has been the most-cited publication till now (Lambert et al., 2013). The sample size of GWAS kept expanding in recent years. In 2021, a meta-analysis study including 1,126,563 individuals identified 38 susceptibility loci for the late-onset form of AD, which was the largest GWAS for clinically diagnosed AD to date (Wightman et al., 2021). So far, around 70 loci implicating the risk for AD have been identified by GWAS, providing strong evidence for the genetic pathogenesis of AD.

Then, we got an overview of GWAS in AD by demonstrating the main contributors and their cooperation networks. The highest number of studies was generated by the United States (775), followed by China (309) and the United Kingdom (302). When ranked by total citations, the United States had an overwhelming 42,237 citations, and the United Kingdom ranked second with 25,311 citations. Although China had many publications, the total citations were not high (7,259) (Table 1). The top ten institutions included nine in the United States and one in China (Table 2). The most influential researchers were Bennett DA, Farrer LA, Mayeux R, De Jager PL, Schellenberg GD, Peterson RC, Haines JL and Younkin SG from the United States, Hardy J from the United Kingdom, and Amouyel P from France (Figure 5A). From the results above, it can be found that the United States was the country that had made a remarkable contribution in this field. China has also explored much, but the research quality needs further improvement. The cooperation analysis indicates that researchers in the United States collaborated extensively with researchers in other countries. While researchers from China preferred to establish cooperative relations with domestic rather than international counterparts (Figure 5B). According to Table 4, *Neurobiology of Aging* was the journal that published the most research in this field, and *Nature Genetics* was the authority of this field with the largest number of citations.

As indicated in Figure 6B, after clustering the co-cited publications, the knowledge base map was generated by extracting words from publication titles. The knowledge bases included #polygenic risk scores, #risk variant, #*TREM2*, #meta-analysis, #*PICALM*, #eQTLs, #snRNA-seq, #linkage analysis, #microglia and #Mendelian randomization, which were important words or phrases that summarize the basic concepts underlying the field, including research objects and methods of GWAS in AD.

Knowledge flow analysis is an intuitive way to show the developmental trajectory of research. The cited literature was published earlier, and the citing literature was published later. As Figure 7 demonstrates, the studies were mainly published in journals of basic research (molecular, biology, and genetics) in earlier times. Later studies were published in both journals of basic research (molecular, biology, immunology) and journals of clinical medicine (medicine, medical clinical), reflecting a shift in the research focus from bench to practice over the years.

### Research hotspots of AD pathogenesis

#### Aβ and tau: pathological biomarkers and the genetic basis

Our keyword analysis revealed that #Aβ and #tau were frequently mentioned keywords in this field. Extracellular plaques that consist of Aβ and intracellular neurofibrillary tangles (NFT) with over-phosphorylated tau protein are the basic pathological features of AD, and are recognized as major players in disease progression (Sierksma et al., 2020).

Aβ was the most well-studied pathological feature, as indicated by Figure 8A. It had a research fever in recent years (a citation burst from 2020 to 2022, Figure 8B), indicating its core position in AD genetic studies. In Aβ pathology, altered amyloid precursor protein (APP) metabolism with the overproduction of Aβ peptides is the primary cause of amyloidosis, and *APOE*ε*4* and *ABCA7* were identified to be most strongly associated with Aβ production (Apostolova et al., 2018). Of all the APOE protein isoforms, APOEε4 has the strongest effect on stimulating neuronal Aβ production by enhancing *APP* transcription (Huang Y. W. A. et al., 2017). *ABCA7* loss of function facilitates the process of APP protein cleaving, leading to rapid accumulation of cortical Aβ at the early stage of AD (Sakae et al., 2016). Moreover, several GWAS-defined genes linked to endocytosis (*BIN1*, *CD2AP* and *PICALM*) and endosomes (*FERMT2* and *SORL1*) can also modulate APP metabolism and Aβ production (Dourlen et al., 2019). The dysregulated clearance pathway is another cause of Aβ accumulation. In normal conditions, LRP1 receptor in neurons is shown to uptake Aβ-APOE complexes. But when the *APOEε4* variant takes place, the clearance rate is lower. At the same time, *PICALM* regulates Aβ clearance across the blood–brain barrier by internalization and transcytosis, and the variants of *PICALM* disrupt the mechanism (Zhao et al., 2015).

Tau is another essential feature of AD progression. The prevalent notion is that pathological tau is integral for Aβ to exert neurotoxicity (Frost, 2023). A recent study has strongly suggested that tau protein is also an early indicator of preclinical AD, that aggregations of tau protein seem to precede the deposition of Aβ by approximately a decade, and that the location of pathogenic tau, but not Aβ, can predict the degeneration of the brain areas in the following 2 years (La Joie et al., 2020). Thus, uncovering the genetic factor associated with tau pathology is particularly important for intervening in the disease process in the preclinical stage. *BIN1*, the first tau-related AD risk gene, has been the focus of tau pathology. Compiling GWAS evidence indicated that variants in *BIN1* increase AD risk (Kingwell, 2013; Franzmeier et al., 2019), and its variants have been proven to be associated with only tau loads but not Aβ loads in AD brains (Chapuis et al., 2013). Several mechanism studies have reported the protective role of *BIN1*. A study using transgenic mice concludes that *BIN1* overexpression prevents both tau mislocalization and somatic inclusion in the hippocampus and directly binds tau to rescue long-term memory deficits (Sartori et al., 2019). Lower *BIN1* levels promote tau propagation by efficiently increasing endocytosis and endosomal trafficking (Calafate et al., 2016). However, there are opposite opinions that loss of forebrain *BIN1* mitigates tau pathology in the hippocampus and entorhinal/piriform cortex of the tauopathy mice, thus attenuating synapse loss, neuronal death, neuroinflammation and brain atrophy (Ponnusamy et al., 2023). Other genes were also reported to affect tau pathology. *APOEε4* increases tau phosphorylation and aggregation (Therriault et al., 2020), and *PTK2B* acts as a tau toxicity suppressor (Brody et al., 2022). Although an increasing number of functional studies demonstrated that GWAS-identified AD risk genes are associated with tau pathology, the tau-related genetic profile is less uncovered compared to Aβ.

In recent years, GWAS based on neuroimaging or cerebrospinal fluid (CSF) Aβ and tau levels has been conducted. Although in its initial stage, it has identified vital genes associated with Aβ and tau (Li et al., 2022). Ramanan et al. conducted the first neuroimaging GWAS by integrating positron emission tomography (PET) phenotypes with genetic data, confirming the association of *APOE* and *BCHE* with Aβ burden (Ramanan et al., 2014). In the latest GWAS of tau-PET, the *APOE* dysfunction is emphasized, and novel loci regulating *VNN2* and *EYA4* are identified (Guo Y. et al., 2022). At present, the sample sizes are relatively small, and large-scale GWAS in AD biomarkers is still lacking.

#### Immunity and microglia: from genetic implications to clinical conversion

In the field of GWAS in AD, #immunity and #microglia were recurring keywords, suggesting the hotspots of AD mechanism (Figure 8A). It has been reported that the enhancer sequences for the immune process are regions where AD risk loci are preferentially enriched (Gjoneska et al., 2015). Over the years, plenty of GWAS-defined genes participating in the immune process (*APOE*, *TREM2*, *CD33*, *CLU*, *CR1*, *ABCA7*, *EPHA1*, *MS4A*s, *INPP5D*, *SPI1*, *PLCG2*, *ABI3*, *HLA-DR15*, etc.) have been reported to be associated to AD (Lambert et al., 2009; Seshadri et al., 2010; Hollingworth et al., 2011; Reitz et al., 2013; Tan et al., 2013; Deming et al., 2017; Huang K. L. et al., 2017; Sims et al., 2017; Kunkle et al., 2019). Among these genes, *TREM2*, *CD33* and *CLU* were the most studied ones according to Figure 8A. This evidence suggests that immunity is a vital part of AD pathogenesis.

As the most important cells of brain immune surveillance and neuronal support (Chen and Holtzman, 2022), microglia were also the research focus and have become increasingly popular with a citation burst after 2021 in the field (Figures 8A,B). Microglia protects the brain by phagocytosis, Aβ degradation and anti-inflammatory cytokines secretion under homeostatic conditions, while microglial excessive activation and phenotype conversion in AD brains leads to the release of pro-inflammatory cytokines and complement (Leng and Edison, 2021). Nott et al. identify that common non-coding variants associated with AD risk are enriched in microglial enhancers (Nott et al., 2019). Many AD risk genes involved in immune response and phagocytic function are highly expressed by microglia (Efthymiou and Goate, 2017), suggesting that microglia is the gathering spot of immune function.

The triggering receptor expressed on myeloid cell 2 (TREM2) is a microglial receptor encoded by *TREM2*. It is pivotal for maintaining the microglial cell number and function (Zheng et al., 2016). *TREM2* R47H variant has been identified as a risk factor for AD as early as 2013 (Guerreiro et al., 2013), while mechanism studies have implicated that the unwanted mutations in *TREM2* can lead to the reduced protective sTREM2 release, diminished microglial viability, and subsequent increased Aβ burden and neuroinflammation (Carmona et al., 2018; Zhao et al., 2018). In contrast to the protective role of *TREM2*, evidence suggests that *CD33* inhibits microglial phagocytosis function and promotes pro-inflammatory microglia activation. CD33-immunoreactive microglia are positively correlated with plaque burden in AD brains, and the overexpression of *CD33* may lead to inhibited Aβ uptake (Griciuc et al., 2013). Interestingly, TREM2 can act as a downstream switch to turn off the pro-inflammatory effects of CD33 (Griciuc et al., 2019).

Evidence from abundant gene-level studies put *TREM2* in the spotlight for immunotherapy. A study by van Lengerich B et al. reported that a TREM2-activating antibody is capable of promoting microglial glucose metabolism in AD mice (van Lengerich et al., 2023). Wang et al. established a microglia-targeted gene delivery system for the treatment of AD and successfully remodeled dysfunctional microglia by TREM2 (Wang et al., 2022). Zhao et al. identified and modified a TREM2 agonist monoclonal Ab18 that can greatly enhance TREM2 activation. The modified-Ab18 treatment can significantly increase microglial phagocytosis of Aβ, reduce tau protein and improve cognition of AD mice (Zhao et al., 2022). The latest study by Yoo et al. using a *TREM2* mutant AD mouse model suggests that microglial function can be restored by systemic hematopoietic cell transplantation followed by microglia replacement (Yoo et al., 2023). These studies provide the possibility for clinical conversion of immunotherapy targeting *TREM2*.

*CR1* and *CLU* of the complement system also participate in brain immunity. *CR1* encodes a complement regulatory protein with both beneficial and detrimental effects. Higher *CR1* expression removes C3b-Aβ immune complexes and lowers Aβ load (Li et al., 2021). However, *CR1* overexpression also leads to neuron damage (Crehan et al., 2013), and the closest ortholog of *CR1* (*Crry*) silencing leads to reduced tau pathology (Zhu et al., 2022). The other gene, *CLU,* also regulates Aβ in several dimensions. Its protein product clusterin interferes with Aβ aggregation by binding with oligomers, regulating Aβ microglial uptake, and modulating Aβ transport through the blood–brain barrier. However, whether clusterin-Aβ aggregation is more toxic than aggregates containing Aβ alone is controversial, and whether clusterin can promote Aβ clearance is still unknown (Foster et al., 2019). Although GWAS have suggested that *CR1* and *CLU* are strongly associated with AD, the biological or pathological functions of *CR1* and *CLU* in the pathogenesis of AD are far from clear.

The accumulating genetic evidence by GWAS emphasizes the preponderant role of immunity and microglia in the pathogenesis of AD, which has aroused researchers’ interest in mechanism studies concerning AD and immunity. Therefore, we can speculate that immunotherapies may become a promising research direction after the failure of AD therapies targeting Aβ and tau.

#### DNA methylation is also one of the hotspots

DNA methylation is the most studied epigenetic mechanism of AD, and detecting DNA methylation by epigenomics has become a branch of AD genetic study. The degree of DNA methylation greatly affects gene expression. DNA hypermethylation often downregulates gene expression, while DNA hypomethylation increases gene expression and functional activation (Younesian et al., 2022). In AD pathology, Aβ related genes such as *APP*, *PSEN1*, and *BACE1* tend to be DNA hypomethylated (Schrötter et al., 2012), directly resulting in Aβ plaque overload (De Jager et al., 2014). Besides, several genes are found to be hypermethylated in AD, including *ANK1*, *RPL13*, *RHBDF2*, *DUSP22*, *SORL1*, *ABCA7*, *BIN1*, *SLC24A4*, *HLA-DRB5*, *HOXA*, which are mainly related to Aβ deposition (Lunnon et al., 2014; Yu et al., 2015; Smith et al., 2018; Min et al., 2021; Nabais et al., 2021). Nowadays, research on epigenomics has become a research hotspot, and studies committed to discovering new DNA methylation sites are rising.

### Research fronts in GWAS for the AD field

Figure 8B manifested keywords with a citation burst in recent years (with its burst time followed): Aβ (2020–2022), UK Biobank (2020–2022), meta-analysis (2020–2022), causal association (2020–2022), causal effect (2020–2022), disease (2020–2022), microglia (2021–2022) and sensitivity analysis (2021–2022), and these keywords indicated the research fronts and future trends of the GWAS in AD field.

#### Disease (2020–2022)

Diseases or syndromes that have similarities to AD have become the research focus in recent years. According to Figure 8A, diseases including Parkinson’s disease (PD) and schizophrenia, and syndrome including mild cognitive impairment (MCI), were common keywords in GWAS for the AD field, suggesting that these diseases or syndrome may partially overlap with genetic features of AD, and searching for genetic similarities of these diseases has become a popular research direction.

MCI due to AD is a stage between healthy aging and dementia, and genetic evidence supports the view that its conversion into dementia is primarily due to the AD pathway. Thus, MCI and AD are often studied in one GWAS as two subgroups (Adams et al., 2015). PD and AD are both neurodegenerative diseases, and although the clinical presentations are different, finding the genetic similarity of these two types of neurodegenerative diseases has become a new research focus. A large multi-center study identifies that Lewy body dementia shares risk genes and pathways with AD and PD (Chia et al., 2021; Guo P. et al., 2022). Recently, Reginal and colleagues observed a significant local genetic correlation between AD and PD in the *PBK* and *SCARA5* genic regions (Reynolds et al., 2023). Meanwhile, Raffaele et al. reported SNPs within *HLA*, *MAPT* and *APOE* regions jointly contributing to increased risk for AD or PD (Ferrari et al., 2017). In addition, schizophrenia is also investigated considering psychotic symptoms are present in approximately 50% of AD individuals (Murray et al., 2014). Several SNPs are reported to show the same effect on schizophrenia and AD with psychotic symptoms, indicating the genetic connectivity between AD and schizophrenia (Alfimova et al., 2019).

#### Causal association (2020–2022) and causal effect (2020–2022)

GWAS uncovers the association of many genetic loci with traits and diseases. However, even the variants most strongly associated with AD are not necessarily causal (Andrews et al., 2020). In recent years, pinpointing AD causal risk genes has been a trendy topic. The development of fine mapping and gene prioritization enables researchers to determine whether the loci-trait is causal to the phenotype (Broekema et al., 2020). Amlie-Wolf et al. applied fine-mapping approaches in their study and identified candidate causal variants in four genes: *EPHA1*, *CD33*, *BIN1* and *CD2AP* (Amlie-Wolf et al., 2018). Corces et al. nominates multiple AD risk variants that may be causal, including variants in the *MS4A* and *BIN1* loci (Corces et al., 2020). Recent research by Schwartzentruber suggests that *CCDC6*, *TSPAN14*, *NCK2*, *SPRED2*, *BIN1*, *APH1B*, *PTK2B*, *PILRA* and *CASS4* are likely causal genes of AD (Schwartzentruber et al., 2021). Not much research has been done on causal variants of AD so far, and there is still a long way to go.

#### Other research fronts

UK Biobank (2020–2022) is a biomedical database containing half a million volunteers’ genetic and health information (Littlejohns et al., 2020). UK Biobank is the basis of many large-scale GWAS due to its unparalleled resources, significantly prompting AD genetic research. UK Biobank samples are almost entirely from the United Kingdom, which reflects that GWAS are based on specific regions and ethnicities. To some extent, European ancestry is overrepresented in genetic studies, and data based on other regions or ethnicities are relatively scarce, despite African Americans and Hispanic Americans being more likely to develop AD in the same community (Andrews et al., 2023).

As GWAS continues to evolve, some methodologies have also been developed. Mendelian randomization (2020–2022) is a statistical method based on GWAS, which is used to infer causal associations between exposures and disease outcomes using genetic variants. Mendelian randomization can effectively reduce confounding bias due to the advantage of alleles being randomly assigned to offspring. Meanwhile, sensitivity analysis (2021–2022) is a way to assess the reliability of conclusions obtained from Mendelian randomization studies and further enhance the studies’ credibility. In recent years, Mendelian randomization and sensitivity analysis have become cutting-edge methods frequently used in AD genetic studies.

### Limitations

There are certain limitations to our study. Only references in the WoSCC database and publications written in English were included in our study owing to the nature of bibliometric software. The publications we included in this work were selected by database retrieval as well as manual screening, and therefore have an unavoidable subjective judgment bias. Although we covered a majority of publications, the study cannot fully describe the bibliometric information of the field.

## Conclusion

This bibliometric and visualization study demonstrates what GWAS in AD is like, what it tells us, and where it is headed. According to our bibliometric analysis, the most concerned AD pathogenesis and current research hotspots were (1) Aβ and tau, (2) immunity and microglia, with *TREM2* as a potential immunotherapy target, and (3) DNA methylation. The important pathogenesis implied by genetic studies is valuable for future mechanism studies and clinical conversion. The research fronts were (1) looking for genetic similarities between AD and other neurological diseases and syndromes, and (2) searching for causal variants of AD. Current weaknesses of the discipline are (1) GWAS in AD biomarkers based on relatively large sample sizes are scarce, (2) studies of causal variants of AD are few, and (3) GWAS in AD based on non-European populations are inadequate. It is necessary to pay more attention to these under-researched directions in the future. Our study provides a comprehensive view of GWAS in AD. Researchers new to the field can easily obtain useful information from our research and better investigate the genetic etiology of AD.

## Data availability statement

The original contributions presented in the study are included in the article/Supplementary material, further inquiries can be directed to the corresponding author.

## Author contributions

JZ: Conceptualization, Data curation, Methodology, Software, Visualization, Writing – original draft, Writing – review & editing. YW: Data curation, Methodology, Writing – original draft. YZ: Data curation, Methodology, Writing – review & editing. JY: Conceptualization, Funding acquisition, Investigation, Writing – review & editing.

## References

[ref1] Adams H. H. H. De Bruijn R. F. A. G. Hofman A. Uitterlinden A. G. Van Duijn C. M. Vernooij M. W.. (2015). Genetic risk of neurodegenerative diseases is associated with mild cognitive impairment and conversion to dementia. Alzheimers Dement. 11, 1277–1285. doi: 10.1016/j.jalz.2014.12.008 25916564

[ref2] Alfimova M. Kondratyev N. Golov A. Golimbet V. (2019). Relationship between Alzheimer's disease-associated Snps within the Clu gene, local Dna methylation and episodic verbal memory in healthy and schizophrenia subjects. Psychiatry Res. 272, 380–386. doi: 10.1016/j.psychres.2018.12.134 30599442

[ref3] Amlie-Wolf A. Tang M. Mlynarski E. E. Kuksa P. P. Valladares O. Katanic Z.. (2018). Inferno: inferring the molecular mechanisms of noncoding genetic variants. Nucleic Acids Res. 46, 8740–8753. doi: 10.1093/nar/gky686, PMID: 30113658 PMC6158604

[ref4] Andrews S. J. Fulton-Howard B. Goate A. (2020). Interpretation of risk loci from genome-wide association studies of Alzheimer's disease. Lancet 19, 326–335. doi: 10.1016/S1474-4422(19)30435-1, PMID: 31986256 PMC8176461

[ref5] Andrews S. J. Renton A. E. Fulton-Howard B. Podlesny-Drabiniok A. Marcora E. Goate A. M. (2023). The complex genetic architecture of Alzheimer's disease: novel insights and future directions. EBioMedicine 90:104511. doi: 10.1016/j.ebiom.2023.104511, PMID: 36907103 PMC10024184

[ref6] Anonymous (2023). 2023 Alzheimer's disease facts and figures. Alzheimers Dement. 19, 1598–1695. doi: 10.1002/alz.13016, PMID: 36918389

[ref7] Apostolova L. G. Risacher S. L. Duran T. Stage E. C. Goukasian N. West J. D.. (2018). Associations of the top 20 Alzheimer Disease risk variants with brain amyloidosis. JAMA Neurol. 75, 328–341. doi: 10.1001/jamaneurol.2017.4198, PMID: 29340569 PMC5885860

[ref8] Ayodele T. Rogaeva E. Kurup J. T. Beecham G. Reitz C. (2021). Early-onset Alzheimer's Disease: what is missing in research? Curr. Neurol. Neurosci. Rep. 21:4. doi: 10.1007/s11910-020-01090-y 33464407 PMC7815616

[ref9] Bertram L. Lill C. M. Tanzi R. E. (2010). The genetics of Alzheimer disease: back to the future. Neuron 68, 270–281. doi: 10.1016/j.neuron.2010.10.013 20955934

[ref10] Bertram L. Mcqueen M. B. Mullin K. Blacker D. Tanzi R. E. (2007). Systematic meta-analyses of Alzheimer disease genetic association studies: the AlzGene database. Nat. Genet. 39, 17–23. doi: 10.1038/ng1934 17192785

[ref11] Brody A. H. Nies S. H. Guan F. Smith L. M. Mukherjee B. Salazar S. A.. (2022). Alzheimer risk gene product Pyk2 suppresses tau phosphorylation and phenotypic effects of tauopathy. Mol. Neurodegener. 17:32. doi: 10.1186/s13024-022-00526-y, PMID: 35501917 PMC9063299

[ref12] Broekema R. V. Bakker O. B. Jonkers I. H. (2020). A practical view of fine-mapping and gene prioritization in the post-genome-wide association era. Open Biol. 10:190221. doi: 10.1098/rsob.190221, PMID: 31937202 PMC7014684

[ref13] Calafate S. Flavin W. Verstreken P. Moechars D. (2016). Loss of Bin1 promotes the propagation of tau pathology. Cell Rep. 17, 931–940. doi: 10.1016/j.celrep.2016.09.063, PMID: 27760323

[ref14] Carmona S. Zahs K. Wu E. Dakin K. Bras J. Guerreiro R. (2018). The role of Trem2 in Alzheimer's disease and other neurodegenerative disorders. Lancet 17, 721–730. doi: 10.1016/S1474-4422(18)30232-1 30033062

[ref15] Chapuis J. Hansmannel F. Gistelinck M. Mounier A. Van Cauwenberghe C. Kolen K. V.. (2013). Increased expression of Bin1 mediates Alzheimer genetic risk by modulating tau pathology. Mol. Psychiatry 18, 1225–1234. doi: 10.1038/mp.2013.1, PMID: 23399914 PMC3807661

[ref16] Chen C. (2017). Science mapping: a systematic review of the literature. J. Data Inf. Sci. 2, 1–40. doi: 10.1515/jdis-2017-0006

[ref17] Chen X. Holtzman D. M. (2022). Emerging roles of innate and adaptive immunity in Alzheimer's disease. Immunity 55, 2236–2254. doi: 10.1016/j.immuni.2022.10.016, PMID: 36351425 PMC9772134

[ref18] Chia R. Sabir M. S. Bandres-Ciga S. Saez-Atienzar S. Reynolds R. H. Gustavsson E.. (2021). Genome sequencing analysis identifies new loci associated with Lewy body dementia and provides insights into its genetic architecture. Nat. Genet. 53, 294–303. doi: 10.1038/s41588-021-00785-3, PMID: 33589841 PMC7946812

[ref19] Corces M. R. Shcherbina A. Kundu S. Gloudemans M. J. Frésard L. Granja J. M.. (2020). Single-cell epigenomic analyses implicate candidate causal variants at inherited risk loci for Alzheimer's and Parkinson's diseases. Nat. Genet. 52, 1158–1168. doi: 10.1038/s41588-020-00721-x, PMID: 33106633 PMC7606627

[ref20] Crehan H. Hardy J. Pocock J. (2013). Blockage of Cr1 prevents activation of rodent microglia. Neurobiol. Dis. 54, 139–149. doi: 10.1016/j.nbd.2013.02.003, PMID: 23454195

[ref21] De Jager P. L. Srivastava G. Lunnon K. Burgess J. Schalkwyk L. C. Yu L.. (2014). Alzheimer's disease: early alterations in brain Dna methylation at Ank1, Bin1, Rhbdf2 and other loci. Nat. Neurosci. 17, 1156–1163. doi: 10.1038/nn.3786, PMID: 25129075 PMC4292795

[ref22] Deming Y. Li Z. Kapoor M. Harari O. Del-Aguila J. L. Black K.. (2017). Genome-wide association study identifies four novel loci associated with Alzheimer's endophenotypes and disease modifiers. Acta Neuropathol. 133, 839–856. doi: 10.1007/s00401-017-1685-y, PMID: 28247064 PMC5613285

[ref23] Dourlen P. Kilinc D. Malmanche N. Chapuis J. Lambert J.-C. (2019). The new genetic landscape of Alzheimer's disease: from amyloid cascade to genetically driven synaptic failure hypothesis? Acta Neuropathol. 138, 221–236. doi: 10.1007/s00401-019-02004-0, PMID: 30982098 PMC6660578

[ref24] Efthymiou A. G. Goate A. M. (2017). Late onset Alzheimer's disease genetics implicates microglial pathways in disease risk. Mol. Neurodegener. 12:43. doi: 10.1186/s13024-017-0184-x, PMID: 28549481 PMC5446752

[ref25] Ferrari R. Wang Y. Vandrovcova J. Guelfi S. Witeolar A. Karch C. M.. (2017). Genetic architecture of sporadic frontotemporal dementia and overlap with Alzheimer's and Parkinson's diseases. J. Neurol. Neurosurg. Psychiatry 88, 152–164. doi: 10.1136/jnnp-2016-314411, PMID: 27899424 PMC5237405

[ref26] Foster E. M. Dangla-Valls A. Lovestone S. Ribe E. M. Buckley N. J. (2019). Clusterin in Alzheimer's Disease: mechanisms, Genetics, and lessons from other pathologies. Front. Neurosci. 13:164. doi: 10.3389/fnins.2019.00164, PMID: 30872998 PMC6403191

[ref27] Franzmeier N. Rubinski A. Neitzel J. Ewers M. (2019). The Bin1 rs744373 Snp is associated with increased tau-pet levels and impaired memory. Nat. Commun. 10:1766. doi: 10.1038/s41467-019-09564-5 30992433 PMC6467911

[ref28] Frost B. (2023). Alzheimer's disease and related tauopathies: disorders of disrupted neuronal identity. Trends Neurosci. 46, 797–813. doi: 10.1016/j.tins.2023.07.006, PMID: 37591720 PMC10528597

[ref29] Gibson G. (2010). Hints of hidden heritability in Gwas. Nat. Genet. 42, 558–560. doi: 10.1038/ng0710-558 20581876

[ref30] Gjoneska E. Pfenning A. R. Mathys H. Quon G. Kundaje A. Tsai L.-H.. (2015). Conserved epigenomic signals in mice and humans reveal immune basis of Alzheimer's disease. Nature 518, 365–369. doi: 10.1038/nature14252 25693568 PMC4530583

[ref31] Golde T. E. (2022). Disease-modifying therapies for Alzheimer's Disease: more questions than answers. Neurotherapeutics 19, 209–227. doi: 10.1007/s13311-022-01201-2, PMID: 35229269 PMC8885119

[ref32] Griciuc A. Patel S. Federico A. N. Choi S. H. Innes B. J. Oram M. K.. (2019). Trem2 acts downstream of Cd33 in modulating microglial pathology in Alzheimer's Disease. Neuron 103, 820–835.e7. doi: 10.1016/j.neuron.2019.06.010, PMID: 31301936 PMC6728215

[ref33] Griciuc A. Serrano-Pozo A. Parrado A. R. Lesinski A. N. Asselin C. N. Mullin K.. (2013). Alzheimer's disease risk gene Cd33 inhibits microglial uptake of amyloid beta. Neuron 78, 631–643. doi: 10.1016/j.neuron.2013.04.014, PMID: 23623698 PMC3706457

[ref34] Guerreiro R. Wojtas A. Bras J. Carrasquillo M. Rogaeva E. Majounie E.. (2013). Trem2 variants in Alzheimer's disease. N. Engl. J. Med. 368, 117–127. doi: 10.1056/NEJMoa1211851 23150934 PMC3631573

[ref35] Guo P. Gong W. Li Y. Liu L. Yan R. Wang Y.. (2022). Pinpointing novel risk loci for Lewy body dementia and the shared genetic etiology with Alzheimer's disease and Parkinson's disease: a large-scale multi-trait association analysis. BMC Med. 20:214. doi: 10.1186/s12916-022-02404-2, PMID: 35729600 PMC9214990

[ref36] Guo Y. Yang Y.-X. Zhang Y.-R. Huang Y.-Y. Chen K.-L. Chen S.-D.. (2022). Genome-wide association study of brain tau deposition as measured by 18F-flortaucipir positron emission tomography imaging. Neurobiol. Aging 120, 128–136. doi: 10.1016/j.neurobiolaging.2022.09.002, PMID: 36195041

[ref37] Harold D. Abraham R. Hollingworth P. Sims R. Gerrish A. Hamshere M. L.. (2009). Genome-wide association study identifies variants at Clu and Picalm associated with Alzheimer's disease. Nat. Genet. 41, 1088–1093. doi: 10.1038/ng.440, PMID: 19734902 PMC2845877

[ref38] Hollingworth P. Harold D. Sims R. Gerrish A. Lambert J.-C. Carrasquillo M. M.. (2011). Common variants at Abca7, Ms4A6A/Ms4A4E, Epha1, Cd33 and Cd2ap are associated with Alzheimer's disease. Nat. Genet. 43, 429–435. doi: 10.1038/ng.803, PMID: 21460840 PMC3084173

[ref39] Hsiao Y.-H. Chang C.-H. Gean P.-W. (2018). Impact of social relationships on Alzheimer's memory impairment: mechanistic studies. J. Biomed. Sci. 25:3. doi: 10.1186/s12929-018-0404-x, PMID: 29325565 PMC5764000

[ref40] Huang K. L. Marcora E. Pimenova A. A. Di Narzo A. F. Kapoor M. Jin S. C.. (2017). A common haplotype lowers Pu.1 expression in myeloid cells and delays onset of Alzheimer's disease. Nat. Neurosci. 20, 1052–1061. doi: 10.1038/nn.4587, PMID: 28628103 PMC5759334

[ref41] Huang Y. W. A. Zhou B. Wernig M. Südhof T. C. (2017). ApoE2, ApoE3, and ApoE4 differentially stimulate app transcription and Aβ secretion. Cells 168, 427–441.e21. doi: 10.1016/j.cell.2016.12.044, PMID: 28111074 PMC5310835

[ref42] Ikram M. A. Decarli C. (2012). Next frontiers in the genetic epidemiology of Alzheimer's disease. Eur. J. Epidemiol. 27, 831–836. doi: 10.1007/s10654-012-9742-2, PMID: 23132737 PMC3774602

[ref43] Jansen I. E. Savage J. E. Watanabe K. Bryois J. Williams D. M. Steinberg S.. (2019). Genome-wide meta-analysis identifies new loci and functional pathways influencing Alzheimer's disease risk. Nat. Genet. 51, 404–413. doi: 10.1038/s41588-018-0311-9, PMID: 30617256 PMC6836675

[ref9001] Jun G Ibrahim-Verbaas CA Vronskaya M. (2016). A novel Alzheimer disease locus located near the gene encoding tau protein. Mol Psychiatry. 21:108–117. doi: 10.1038/mp.2015.23 25778476 PMC4573764

[ref44] Karch C. M. Goate A. M. (2015). Alzheimer's disease risk genes and mechanisms of disease pathogenesis. Biol. Psychiatry 77, 43–51. doi: 10.1016/j.biopsych.2014.05.006, PMID: 24951455 PMC4234692

[ref45] Khani M. Gibbons E. Bras J. Guerreiro R. (2022). Challenge accepted: uncovering the role of rare genetic variants in Alzheimer's disease. Mol. Neurodegener. 17:3. doi: 10.1186/s13024-021-00505-9, PMID: 35000612 PMC8744312

[ref46] Kingwell K. (2013). Alzheimer disease: Bin1 variant increases risk of Alzheimer disease through tau. Nat. Rev. Neurol. 9:184. doi: 10.1038/nrneurol.2013.34, PMID: 23458971

[ref47] Kunkle B. W. Grenier-Boley B. Sims R. Bis J. C. Damotte V. Naj A. C.. (2019). Genetic meta-analysis of diagnosed Alzheimer's disease identifies new risk loci and implicates Aβ, tau, immunity and lipid processing. Nat. Genet. 51, 414–430. doi: 10.1038/s41588-019-0358-2, PMID: 30820047 PMC6463297

[ref48] La Joie R. Visani A. V. Baker S. L. Brown J. A. Bourakova V. Cha J.. (2020). Prospective longitudinal atrophy in Alzheimer's disease correlates with the intensity and topography of baseline tau-PET. Sci. Transl. Med. 12:eaau5732. doi: 10.1126/scitranslmed.aau5732 31894103 PMC7035952

[ref49] Lambert J.-C. Heath S. Even G. Campion D. Sleegers K. Hiltunen M.. (2009). Genome-wide association study identifies variants at Clu and Cr1 associated with Alzheimer's disease. Nat. Genet. 41, 1094–1099. doi: 10.1038/ng.439 19734903

[ref50] Lambert J. C. Ibrahim-Verbaas C. A. Harold D. Naj A. C. Sims R. Bellenguez C.. (2013). Meta-analysis of 74,046 individuals identifies 11 new susceptibility loci for Alzheimer's disease. Nat. Genet. 45, 1452–1458. doi: 10.1038/ng.2802, PMID: 24162737 PMC3896259

[ref51] Leng F. Edison P. (2021). Neuroinflammation and microglial activation in Alzheimer disease: where do we go from here? Nature reviews. Neurology 17, 157–172. doi: 10.1038/s41582-020-00435-y, PMID: 33318676

[ref53] Li L. Yu X. Sheng C. Jiang X. Zhang Q. Han Y.. (2022). A review of brain imaging biomarker genomics in Alzheimer's disease: implementation and perspectives. Transl. Neurodegener. 11:42. doi: 10.1186/s40035-022-00315-z, PMID: 36109823 PMC9476275

[ref54] Littlejohns T. J. Holliday J. Gibson L. M. Garratt S. Oesingmann N. Alfaro-Almagro F.. (2020). The Uk biobank imaging enhancement of 100,000 participants: rationale, data collection, management and future directions. Nat. Commun. 11:2624. doi: 10.1038/s41467-020-15948-9, PMID: 32457287 PMC7250878

[ref52] Li Y. Laws S. M. Miles L. A. Wiley J. S. Huang X. Masters C. L.. (2021). Genomics of Alzheimer's disease implicates the innate and adaptive immune systems. Cell. Mol. Life Sci. 78, 7397–7426. doi: 10.1007/s00018-021-03986-5, PMID: 34708251 PMC11073066

[ref55] Lunnon K. Smith R. Hannon E. De Jager P. L. Srivastava G. Volta M.. (2014). Methylomic profiling implicates cortical deregulation of Ank1 in Alzheimer's disease. Nat. Neurosci. 17, 1164–1170. doi: 10.1038/nn.3782, PMID: 25129077 PMC4410018

[ref56] Ma R. Ho Y.-S. (2016). Comparison of environmental laws publications in science citation index expanded and social science index: a bibliometric analysis. Scientometrics 109, 227–239. doi: 10.1007/s11192-016-2010-6

[ref9002] Marioni RE McRae AF Bressler J. (2018). Meta-analysis of epigenome-wide association studies of cognitive abilities. Mol Psychiatry. 23:2133–2144. doi: 10.1038/s41380-017-0008-y 29311653 PMC6035894

[ref57] Min J. L. Hemani G. Hannon E. Dekkers K. F. Castillo-Fernandez J. Luijk R.. (2021). Genomic and phenotypic insights from an atlas of genetic effects on Dna methylation. Nat. Genet. 53, 1311–1321. doi: 10.1038/s41588-021-00923-x 34493871 PMC7612069

[ref58] Murray P. S. Kumar S. Demichele-Sweet M. A. A. Sweet R. A. (2014). Psychosis in Alzheimer's disease. Biol. Psychiatry 75, 542–552. doi: 10.1016/j.biopsych.2013.08.020, PMID: 24103379 PMC4036443

[ref59] Nabais M. F. Laws S. M. Lin T. Vallerga C. L. Armstrong N. J. Blair I. P.. (2021). Meta-analysis of genome-wide Dna methylation identifies shared associations across neurodegenerative disorders. Genome Biol. 22:90. doi: 10.1186/s13059-021-02275-5, PMID: 33771206 PMC8004462

[ref60] Naj A. C. Jun G. Beecham G. W. Wang L.-S. Vardarajan B. N. Buros J.. (2011). Common variants at Ms4A4/Ms4A6E, Cd2ap, Cd33 and Epha1 are associated with late-onset Alzheimer's disease. Nat. Genet. 43, 436–441. doi: 10.1038/ng.801, PMID: 21460841 PMC3090745

[ref61] Nott A. Holtman I. R. Coufal N. G. Schlachetzki J. C. M. Yu M. Hu R.. (2019). Brain cell type-specific enhancer-promoter interactome maps and disease-risk association. Science (New York, N.Y.) 366, 1134–1139. doi: 10.1126/science.aay0793, PMID: 31727856 PMC7028213

[ref62] Ponnusamy M. Wang S. Yuksel M. Hansen M. T. Blazier D. M. Mcmillan J. D.. (2023). Loss of forebrain Bin1 attenuates hippocampal pathology and neuroinflammation in a tauopathy model. Brain J. Neurol. 146, 1561–1579. doi: 10.1093/brain/awac318, PMID: 36059072 PMC10319775

[ref63] Ramanan V. K. Risacher S. L. Nho K. Kim S. Swaminathan S. Shen L.. (2014). Apoe and Bche as modulators of cerebral amyloid deposition: a florbetapir pet genome-wide association study. Mol. Psychiatry 19, 351–357. doi: 10.1038/mp.2013.19, PMID: 23419831 PMC3661739

[ref64] Reitz C. Jun G. Naj A. Rajbhandary R. Vardarajan B. N. Wang L.-S.. (2013). Variants in the ATP-binding cassette transporter (ABCA7), apolipoprotein E ϵ4, and the risk of late-onset Alzheimer Disease in African Americans. JAMA 309, 1483–1492. doi: 10.1001/jama.2013.2973, PMID: 23571587 PMC3667653

[ref65] Reynolds R. H. Wagen A. Z. Lona-Durazo F. Scholz S. W. Shoai M. Hardy J.. (2023). Local genetic correlations exist among neurodegenerative and neuropsychiatric diseases. Npj Parkinson's Disease 9:70. doi: 10.1038/s41531-023-00504-1, PMID: 37117178 PMC10147945

[ref66] Sakae N. Liu C.-C. Shinohara M. Frisch-Daiello J. Ma L. Yamazaki Y.. (2016). Abca7 deficiency accelerates amyloid-β generation and Alzheimer's neuronal pathology. J. Neurosci. Off. J. Soc. Neurosci. 36, 3848–3859. doi: 10.1523/JNEUROSCI.3757-15.2016, PMID: 27030769 PMC4812140

[ref67] Sartori M. Mendes T. Desai S. Lasorsa A. Herledan A. Malmanche N.. (2019). Bin1 recovers tauopathy-induced long-term memory deficits in mice and interacts with tau through Thr348 phosphorylation. Acta Neuropathol. 138, 631–652. doi: 10.1007/s00401-019-02017-9 31065832 PMC6778065

[ref68] Schrötter A. Pfeiffer K. El Magraoui F. Platta H. W. Erdmann R. Meyer H. E.. (2012). The amyloid precursor protein (app) family members are key players in S-adenosylmethionine formation by Mat2A and modify Bace1 and Psen1 gene expression-relevance for Alzheimer's disease. Mol. Cell. Proteomics 11, 1274–1288. doi: 10.1074/mcp.M112.019364, PMID: 22879628 PMC3494178

[ref69] Schwartzentruber J. Cooper S. Liu J. Z. Barrio-Hernandez I. Bello E. Kumasaka N.. (2021). Genome-wide meta-analysis, fine-mapping and integrative prioritization implicate new Alzheimer's disease risk genes. Nat. Genet. 53, 392–402. doi: 10.1038/s41588-020-00776-w, PMID: 33589840 PMC7610386

[ref70] Serrano-Pozo A. Das S. Hyman B. T. (2021). Apoe and Alzheimer's disease: advances in genetics, pathophysiology, and therapeutic approaches. Lancet 20, 68–80. doi: 10.1016/S1474-4422(20)30412-9, PMID: 33340485 PMC8096522

[ref71] Seshadri S. Fitzpatrick A. L. Ikram M. A. Destefano A. L. Gudnason V. Boada M.. (2010). Genome-wide analysis of genetic loci associated with Alzheimer disease. JAMA 303, 1832–1840. doi: 10.1001/jama.2010.574, PMID: 20460622 PMC2989531

[ref72] Sierksma A. Lu A. Mancuso R. Fattorelli N. Thrupp N. Salta E.. (2020). Novel Alzheimer risk genes determine the microglia response to amyloid-β but not to tau pathology. EMBO Mol. Med. 12:e10606. doi: 10.15252/emmm.201910606, PMID: 31951107 PMC7059012

[ref73] Sims R. Hill M. Williams J. (2020). The multiplex model of the genetics of Alzheimer's disease. Nat. Neurosci. 23, 311–322. doi: 10.1038/s41593-020-0599-5 32112059

[ref74] Sims R. Van Der Lee S. J. Naj A. C. Bellenguez C. Badarinarayan N. Jakobsdottir J.. (2017). Rare coding variants in Plcg2, Abi3, and Trem2 implicate microglial-mediated innate immunity in Alzheimer's disease. Nat. Genet. 49, 1373–1384. doi: 10.1038/ng.3916, PMID: 28714976 PMC5669039

[ref75] Smith R. G. Hannon E. De Jager P. L. Chibnik L. Lott S. J. Condliffe D.. (2018). Elevated Dna methylation across a 48-kb region spanning the Hoxa gene cluster is associated with Alzheimer's disease neuropathology. Alzheimers Dement. 14, 1580–1588. doi: 10.1016/j.jalz.2018.01.017, PMID: 29550519 PMC6438205

[ref76] Tan L. Yu J.-T. Zhang W. Wu Z.-C. Zhang Q. Liu Q.-Y.. (2013). Association of Gwas-linked loci with late-onset Alzheimer's disease in a northern Han Chinese population. Alzheimers Dement. 9, 546–553. doi: 10.1016/j.jalz.2012.08.007, PMID: 23232270

[ref77] Therriault J. Benedet A. L. Pascoal T. A. Mathotaarachchi S. Chamoun M. Savard M.. (2020). Association of Apolipoprotein E ε4 with medial temporal tau independent of amyloid-β. JAMA Neurol. 77, 470–479. doi: 10.1001/jamaneurol.2019.4421, PMID: 31860000 PMC6990684

[ref78] Van Lengerich B. Zhan L. Xia D. Chan D. Joy D. Park J. I.. (2023). A Trem2-activating antibody with a blood-brain barrier transport vehicle enhances microglial metabolism in Alzheimer's disease models. Nat. Neurosci. 26, 416–429. doi: 10.1038/s41593-022-01240-0, PMID: 36635496 PMC9991924

[ref79] Wang P. Yang P. Qian K. Li Y. Xu S. Meng R.. (2022). Precise gene delivery systems with detachable albumin shell remodeling dysfunctional microglia by Trem2 for treatment of Alzheimer's disease. Biomaterials 281:121360. doi: 10.1016/j.biomaterials.2021.121360 34991033

[ref80] Wightman D. P. Jansen I. E. Savage J. E. Shadrin A. A. Bahrami S. Holland D.. (2021). A genome-wide association study with 1,126,563 individuals identifies new risk loci for Alzheimer's disease. Nat. Genet. 53, 1276–1282. doi: 10.1038/s41588-021-00921-z, PMID: 34493870 PMC10243600

[ref81] Xiao Q. Bai X. Zhang C. He Y. (2022). Advanced high-throughput plant phenotyping techniques for genome-wide association studies: a review. J. Adv. Res. 35, 215–230. doi: 10.1016/j.jare.2021.05.002, PMID: 35003802 PMC8721248

[ref82] Yoo Y. Neumayer G. Shibuya Y. Mader M. M.-D. Wernig M. (2023). A cell therapy approach to restore microglial Trem2 function in a mouse model of Alzheimer's disease. Cell Stem Cell 30:1392. doi: 10.1016/j.stem.2023.08.011, PMID: 37802040

[ref83] Younesian S. Yousefi A.-M. Momeny M. Ghaffari S. H. Bashash D. (2022). The DNA methylation in neurological diseases. Cells 11:3439. doi: 10.3390/cells11213439, PMID: 36359835 PMC9657829

[ref84] Yu L. Chibnik L. B. Srivastava G. P. Pochet N. Yang J. Xu J.. (2015). Association of Brain Dna methylation in Sorl1, Abca7, Hla-Drb5, Slc24A4, and Bin1 with pathological diagnosis of Alzheimer disease. JAMA Neurol. 72, 15–24. doi: 10.1001/jamaneurol.2014.3049, PMID: 25365775 PMC4344367

[ref87] Zhao P. Xu Y. Jiang L. Fan X. Li L. Li X.. (2022). A tetravalent Trem2 agonistic antibody reduced amyloid pathology in a mouse model of Alzheimer's disease. Sci. Transl. Med. 14:eabq0095. doi: 10.1126/scitranslmed.abq0095 36070367

[ref86] Zhao Y. Wu X. Li X. Jiang L.-L. Gui X. Liu Y.. (2018). Trem2 is a receptor for β-amyloid that mediates microglial function. Neuron 97, 1023–1031.e7. doi: 10.1016/j.neuron.2018.01.031, PMID: 29518356 PMC5889092

[ref85] Zhao Z. Sagare A. P. Ma Q. Halliday M. R. Kong P. Kisler K.. (2015). Central role for Picalm in amyloid-β blood-brain barrier transcytosis and clearance. Nat. Neurosci. 18, 978–987. doi: 10.1038/nn.4025, PMID: 26005850 PMC4482781

[ref88] Zheng H. Liu C.-C. Atagi Y. Chen X.-F. Jia L. Yang L.. (2016). Opposing roles of the triggering receptor expressed on myeloid cells 2 and triggering receptor expressed on myeloid cells-like transcript 2 in microglia activation. Neurobiol. Aging 42, 132–141. doi: 10.1016/j.neurobiolaging.2016.03.004, PMID: 27143430 PMC4857884

[ref89] Zhu X.-C. Liu L. Dai W.-Z. Ma T. (2022). Crry silencing alleviates Alzheimer's disease injury by regulating neuroinflammatory cytokines and the complement system. Neural Regen. Res. 17, 1841–1849. doi: 10.4103/1673-5374.332160, PMID: 35017447 PMC8820699

